# Recovery of muscular tissue and functional results of patients treated with a gluteus maximus flap transfer due to chronic abductor deficiency after total hip arthroplasty

**DOI:** 10.1007/s00402-024-05417-z

**Published:** 2024-07-03

**Authors:** Paul Ruckenstuhl, Georgi Wassilew, Katrin Theobald, Christian Hipfl, Matthias Pumberger, Carsten Perka, Sebastian Hardt

**Affiliations:** 1 Center for Musculoskeletal Surgery, Charité University Hospital Berlin, Luisenstraße 64, 10117 Berlin, Germany; 2 Department of Orthopaedics and Trauma, Medical University of Graz, Auenbruggerplatz 5, Graz, A-8036 Austria; 3 Department of Orhtopaedics, University Hospital Greifswald Ferdinand-Sauerbruch-Straße, 17475 Greifswald, Germany

**Keywords:** Abductor mechanism deficiency, Gluteus Maximus flap transfer, Total hip arthroplasty

## Abstract

**Background:**

To evaluate the function of the abductor mechanism after a gluteus maximus flap transfer due to a degeneration of the muscles after hip arthroplasty, we analyzed the post-operative functional outcome as well as radiographic effects in muscle tissue.

**Methods:**

This present study included six consecutive patients operatively treated with a gluteus maximus flap due to chronic gluteal deficiency after total hip arthroplasty. All patients presented a preoperative severe limp, hip abductor deficiency and a history of conservative treatment without the relief of symptoms. MRI scans were performed pre- and postoperatively to evaluate the muscle volume and grade of degeneration of the abductor mechanism. For clinical evaluation, the Harris hip score (HHS) was applied pre- and postoperatively. Moreover, the intensity of pain, the Trendelenburg sign, the internal rotation lag sign and the abductor muscle force were measured before and after surgery.

**Results:**

Overall, the evaluation of the Magnetic Resonance Imaging (MRI) showed no significant changes in total muscle volume during the follow-up period. Separate measurements presented a significant growth of muscle volume for the gluteus minimus and tensor fascia lata compared to preoperative imaging during the follow-up period. The amount of fat volume decreased for all the measured muscles with statistical significance for the gluteus minimus, the gluteus medius and the tensor fascia lata. No further muscle degeneration and no flap necrosis were measured. The postoperative HHS results were not statistically significant compared to the preoperative results.

**Conclusions:**

Besides fair clinical results, the radiological measurements indicate that the flap transfer enables functional muscular tissue recovery and prevents further degeneration. Given these conditions, the gluteus maximus muscle flap transfer represents a viable treatment option for patients with chronic gluteal deficiency in selected patients.

## Introduction

The degeneration of the abductor mechanism is a common cause for patients’ dissatisfaction due to pain, limping and functional limitations [1–3]. Besides the primary disruption and traumatic destruction of the abductor mechanism, total hip arthroplasty (THA) and revision hip arthroplasty remain the most relevant cause for limited abductor function [4, 5]. The diagnosis of abductor lesions can be challenging and is frequently misdiagnosed as postoperative muscle weakness or trochanteric bursitis [6]. Moreover, other causes for abductor insufficiency such as greater trochanter fractures, implant loosening and infection need to be ruled out [7, 8]. Iatrogenic damage to the muscle or to the superior gluteal nerve during the surgical procedure as well as malposition of the implant can cause abductor mechanism deficiency [9, 10]. In the case of intraoperative damage to the gluteus medius muscle, direct re-attachment is recommended [11].

Abductor tears are also related to the surgical approach. Therefore, the direct lateral or anterolateral approach is associated with a higher risk of muscular damage [4, 9, 12]. The anterior approach is limited due to the restricted evaluation of abductor attachment at the greater trochanter. Despite other limiting factors, a carefully performed posterolateral approach seems to lower the risk of damage to the abductor tendons [13, 14]. Nevertheless, clinically significant lesions to the abductor mechanism are known for all optional surgical approaches to the hip joint [15].

Studies have reported an incidence of symptomatic abductor lesions of 25–35% in primary THA [16, 17]. Moreover, considerably higher incidence numbers are known for older, socially deprived patient cohorts, trauma and revision cases as well as for women [5]. With worldwide growing numbers of primary THA followed by an increasing number of revision THA, postoperative complications such as abductor mechanism deficiency are gaining importance. Although the relevance of this pathology is well-known and evident, accurate treatment regimens and knowledge regarding the consequences for the muscular tissue are still lacking. Lesions to the abductor mechanism are often not described or evaluated in THA outcome studies. Therefore, the postoperative degeneration of the abductor mechanism remains one of the most challenging complications in hip surgery.

Theoretically, conservative management including physiotherapy, anti-inflammatory medication, cortico-steroids and lifestyle modification is a feasible treatment option [7, 8]. The effectiveness of conservative treatment is associated with spontaneous improvement, a reluctance of patients to consider further surgery as well as a modification of lifestyle [5]. However, abductor mechanism deficiency after THA is complex and has to be addressed individually [12, 15, 18–20].

Different surgical techniques, including refixation with bone anchors, vastus lateralis advancement, gluteus maximus flap transfer, and allograft reconstruction, are utilized for treating abductor lesions. Postoperative outcomes differ, with no clear advantage noted [20]. Some recent studies presented promising results for the gluteus maximus flap transfer as a specific surgical treatment option [14, 21].

The gluteus maximus flap transfer is a surgical technique that is used to reconstruct predominantly defects of the gluteus medius muscle after total hip arthroplasty. This procedure involves transferring a portion of the gluteus maximus muscle along to repair the tissue defects that may have occurred during the hip arthroplasty surgery. The gluteus maximus muscle plays a vital role in hip extension and lateral rotation of the thigh. Using this muscle for reconstruction can potentially improve the functional outcome [14, 21]. Studies have shown that this technique may improve the stability of the hip joint after surgery and reduce the risk of dislocation [14, 21].

The surgical technique was first described and step-by-step illustrated in 2012 by Whiteside et al. as an alternative approach to address limping, instability, and fatty degeneration of the gluteus medius muscle [18]. The gluteus maximus flap transfer seems to be especially suitable in patients with limited gluteus medius function and chronic abductor tears. Moreover, a sufficient gluteus maximus is required to perform this surgical procedure [18]. However, evidence regarding the interplay of musculature and clinical function is missing; hence, a gold standard does not exist yet [5, 20]. The gluteus muscle flap procedure leads to a modified force transmission of the abductor mechanism [18]. The effect of the surgical procedure on muscle mass and level of degeneration in conjunction with functional results remains unclear and has not yet been investigated.

The purpose of this present study was to examine the success of repair and augmentation with a gluteus maximus transfer for patients suffering from abductor mechanism deficiency by focusing on the functional results as well as on the radiological effects to the muscle tissue. Therefore, first, an evaluation of the postoperative clinical outcome was performed to quantify the success of the treatment. Second, the capacity of adaptation of the new configurated gluteal muscles after surgery was analyzed with MR imaging and third, a focus was set on the interplay between radiological measurements and clinical outcome. To our best knowledge, this is the first study to compare functional results with postoperative MRI in patients treated with a gluteus maximus flap transfer.

## Materials and methods

### Study population

This consecutive case series initially consisted of all patients that were operatively treated with a gluteus maximus flap transfer to address abductor mechanism insufficiency after THA between January 2015 and December 2018 at our institution. In total, 11 patients received surgery during the above-mentioned time. Five patients were excluded for the following reasons: no preoperative MRI (*n* = 2), postoperative periprosthetic fracture (*n* = 1), postoperative death due to other reasons (*n* = 1), and lost to follow-up (*n* = 1). Periprosthetic infection was ruled out by preoperative joint aspiration in all patients before the flap transfer.

The indication for surgery included functional limitation of the abductor mechanism, limping, a positive Trendelenburg sign and hip pain. All patients had failed conservative treatment including physiotherapy, anti-inflammatory medication, cortico-steroids and lifestyle modification, and had exhibited no symptoms relief. The abductor mechanism insufficiency was primarily based on an intraoperative damage to the gluteus medius muscle during hip arthroplasty. All included patients were treated by using a lateral or anterolateral approach to the hip joint. None of the patients reported relevant trauma. A relevant fatty degeneration (≥ 2 Goutallier classification) of the gluteus medius muscle without signs of loosening of the prosthesis was confirmed on preoperative X-rays and MRI scans. All patients were immobilized postoperatively with a unilateral hip cast for six weeks, during which patients were mobilized on crutches with a partial weight-bearing of 15 kg. After six weeks, the cast was removed and patients received a step-by-step guide toward full weight-bearing for an additional six weeks. Afterward, all patients underwent a standardized stationary rehabilitation program. All clinical examinations were performed preoperatively during the admission and postoperatively at the outpatient department.

Two experienced surgeons (G.W. and C.P.) performed all the gluteus maximus transfers of the present study. Surgeries prior to the muscle transfers were performed in different orthopedic departments. Various surgical approaches and implants were utilized.

### Radiographic evaluation

**Fig. 1:**
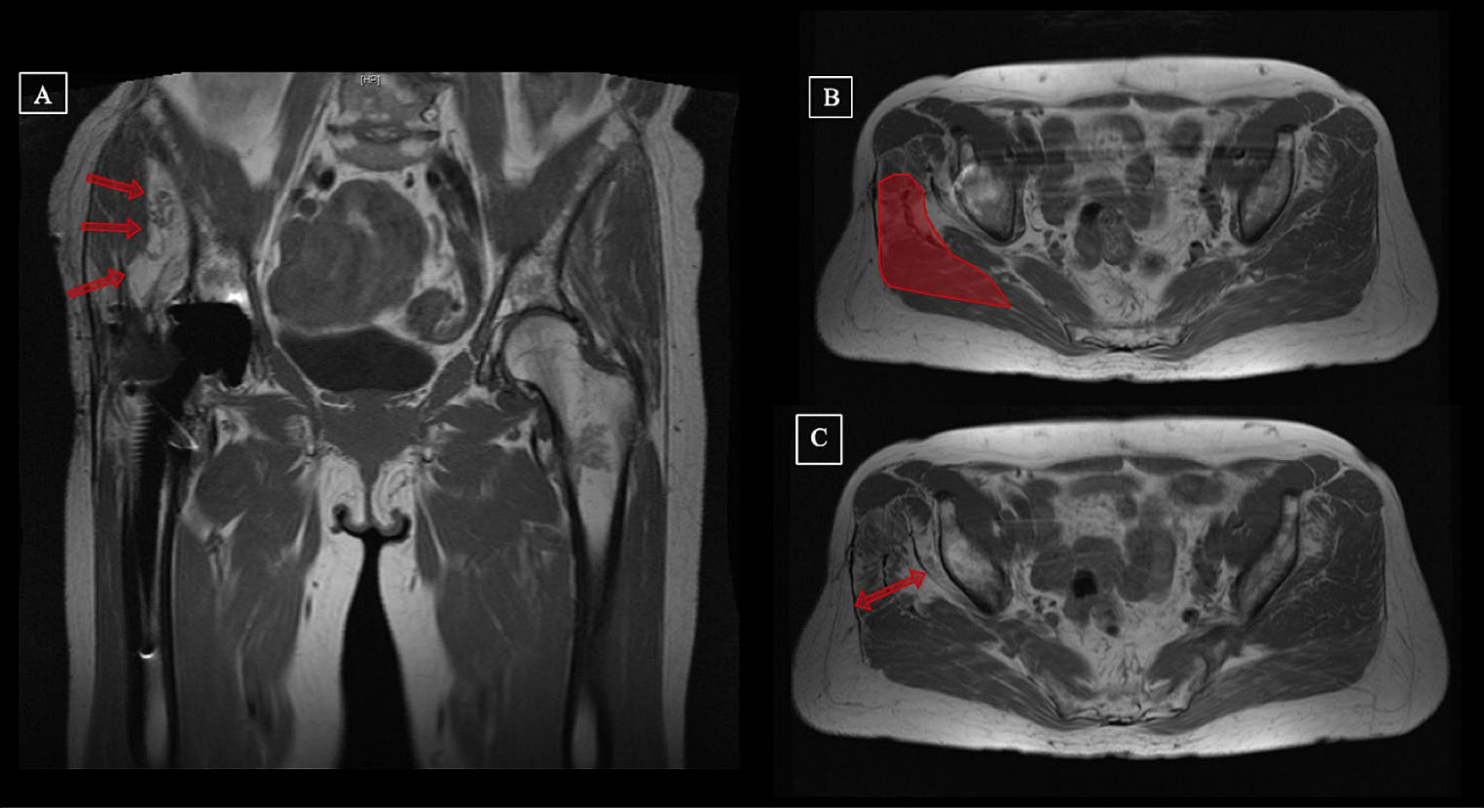
Example and illustration of MRI measurements of the gluteal muscles. Preoperative (**A** and **B**) and postoperative (C and D) MR images after gluteus maximus flap transfer. Measurements regarding the level of degeneration (**A** and **C**, Goutallier IV) and the total muscle volume (**B** and **D**) of the gluteus medius muscle of a 64-year-old patient with chronic abductor mechanism deficiency on the right hip joint after total hip arthroplasty. To receive volumetric results, measurements were performed in slides of the MR image pre- and postoperatively and were highlighted for the gluteus medius muscle in this figure (**B** and **D**)

MRI scans were performed pre- and postoperatively. The total muscle volume, the volume of fatty degenerated muscular tissue, the lean muscle volume (total volume minus fat ratio) and the level of muscular degeneration after the Goutallier classification (G1-G4) were evaluated. All investigations were assessed by a musculoskeletal trained radiologist and an experienced orthopedic surgeon (K.T. and G.E.). Measurements were performed pre- and postoperatively for the following muscles: gluteus minimus (Gmin), gluteus medius (Gmed), gluteus maximus (Gmax) and tensor fascia lata muscle (TFL). The muscle flap size was included in the Gmax volume postoperatively (Fig. 1).

### Clinical evaluation

For clinical evaluation the Harris hip score (HHS) was recorded pre- and postoperatively. Moreover, pain intensity was measured according to the numeric rating scale (NRS 0–10). The muscular force of hip abduction was measured according to Janda´s classification (0–5). The tenderness upon palpation of the greater trochanter, number of prior surgeries, the internal rotation lag sign and the Trendelenburg sign were recorded. The Trendelenburg sign was performed in a standing position on one leg. With a drop in the contralateral hemipelvis of more than two centimeters, the sign was defined as positive [22, 23]. The internal rotation lag sign is a clinical test to indicate abductor tendon lesions [24].

### Magnetic resonance imaging (MRI) and image analysis

All patients were scanned using a MARS-MRI (Metal Artifact Reduction Sequences – Magnetic resonance imaging). For volumetry measurements, the gluteal muscles and the tensor fascia lata muscle were manually segmented from the origin to the insertion of each muscle slice by slice on axial T1-weighted MR-Images by using the Medical Imaging Toolkit (MITK 2018.04.2, DKFZ, Heidelberg, Germany). Three-dimensional muscle volumes were calculated automatically using the program’s interpolation function. Muscular fatty degeneration was assessed with a signal intensity (SI)-based approach by using the subcutaneous fatty tissue as a reference to determine the SI threshold.

### Surgical technique

**Fig. 2:**
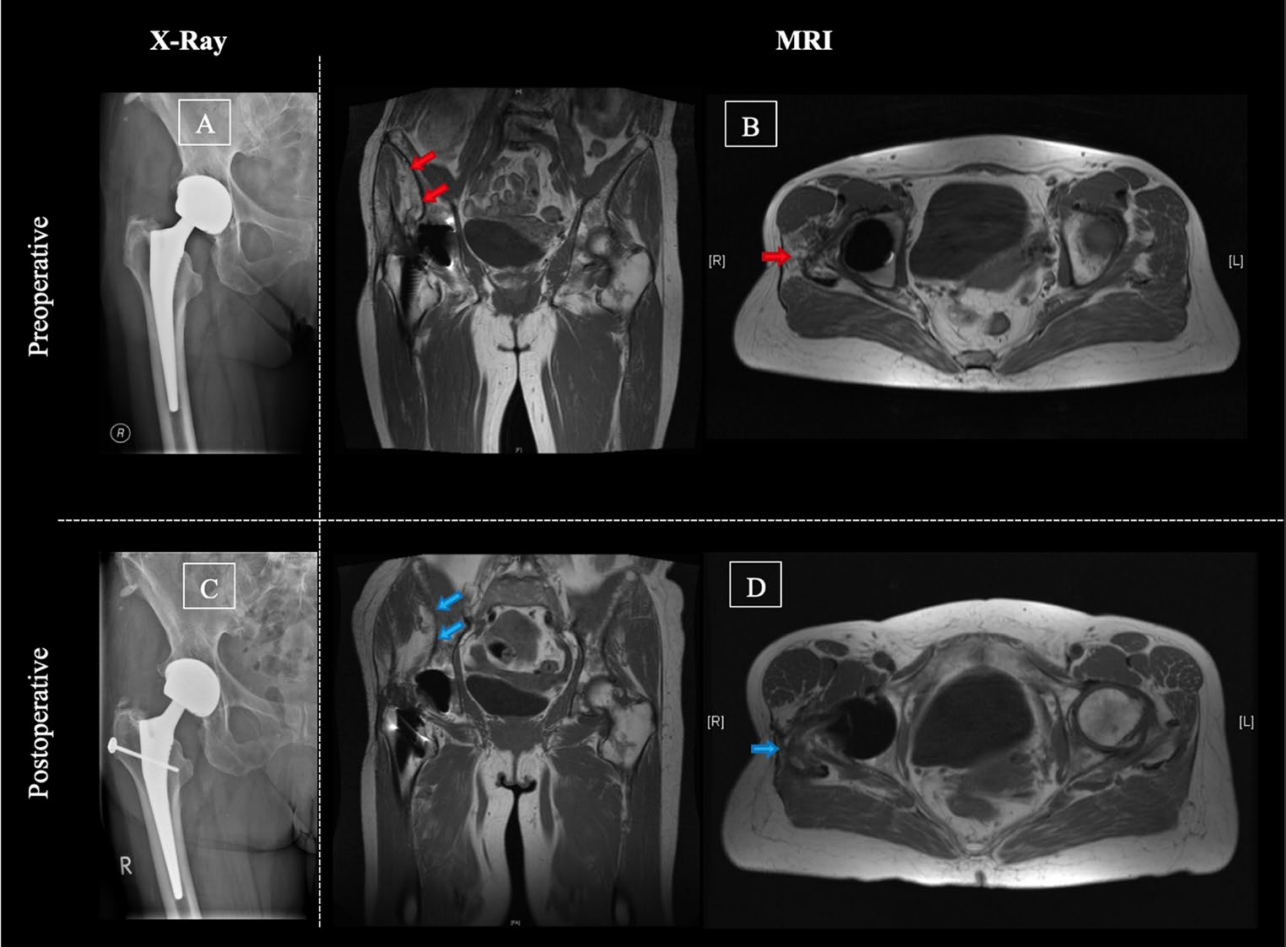
Example of pre- and postoperative radiographic evaluation. Pre- (**A** and **B**) and postoperative (**C** and **D**) X-ray and MRI images of a 69-year-old patient treated with a gluteus maximus muscle flap due to an abductor mechanism deficiency. Preoperatively, a significant degeneration of the gluteus medius muscle (red arrows) was seen without signs of component loosening or infection. The postoperative radiographic images present the screw that was used to fix the anterior flap on the proximal femur (**C**). The postoperative MRI shows no significant further loss of volume or degeneration of the gluteus medius muscle as well as no flap necrosis (**D**, blue arrows)

Leo A. Whiteside published and step-by-step illustrated the surgical technique of the gluteus maximus transfer in 2012 [18]. The technique describes a Gmax flap transfer to compensate for a degenerative modified Gmed and Gmin muscle. Therefore, the gluteus maximus muscle is split in the direction of the muscle fibers towards the fascia latae. This part of the elevated gluteus maximus muscle gets then divided in two flaps. The posterior flap is placed over the femoral neck toward the anterior joint capsule. The anterior flap is positioned directly on the femur distal of the greater trochanter [18]. In our series, the surgical technique for the gluteus maximus flap transfer was marginally modified. Accordingly, a screw and a transosseous fixation was used to fix the tendinous part of the anterior flap distal of the greater trochanter (Fig. 2).

### Harris hip score (HHS)

The HHS is a score to evaluate the results of hip surgeries and applies to different hip disabilities and treatment methods in adult patients [25]. The sore is separated into four sections: pain, function, absence of deformity and range of motion. A maximum of 100 points is reachable. A score of < 70 points is defined as poor, 70–80 as fair, 80–90 as good and 90–100 as excellent.

### Statistical analysis

For statistical analysis IBM SPSS, Version 25, Armonk, NY, IBM Corp was used. Standard descriptive statistics were used to illustrate all baseline and follow-up parameters. Normal distributed data were presented with mean and standard deviation, and for nonparametric data the median was calculated. The statistical significance was evaluated by using the Wilcoxen and McNemar test. The level of significance for all tests was set at *p* < 0.05 and all tests were two- sided. The study was approved by the local Ethics Committee (Nr: EA4/180/18).

All patients provided written informed consent prior to participating in this study and approval was obtained from the local ethics committee. The procedures used in this study adhere to the tenets of the Declaration of Helsinki.

## Results

A total of six patients were included in the present study and were available for clinical and radiographic evaluation at a mean follow-up of 23.5 months (8–36). The population consisted of five females and one male with a mean age of 62 years (50 to 73) at the time of the surgery. The mean BMI was 26.0 (23 to 31). The mean number of prior hip surgeries before the treatment with the Gmax flap was 2.2 (one to four). One patient presented a history of infection with two-staged revision surgery. In two cases, patients had a history of cup revision due to aseptic implant loosening and one patient presented with a history of insufficient gluteus medius refixation. Another two patients presented with postoperative abductor mechanism deficiency after primary hip arthroplasty. In these two cases, the time between primary hip arthroplasty and gluteus maximus transfer was 8 and 18 months, respectively. Overall, the mean time between primary implantation and Gmax transfer was 29.2 (8–43) months. No patient presented a history of joint infection during the last 12 months or a history of instability with hip dislocation (Table 1).

**Table 1:** Patients’ demographics prior to the gluteus medius flap transfer

*n*	Sex (Male/Female)	Age (years)	BMI (kg/m^2^)	FU (months)	Previous surgeries	PI to GMT (months)	Reason for prior revision	Surgical approach
1	F	50	23	10	2	14	ASL	AL
2	F	73	24	36	3	77	ASL	L
3	M	56	23	8	1	8	O	L
4	F	69	31	34	4	43	PPI	AL
5	F	64	24	23	2	12	MR	AL
6	F	69	30	30	1	21	O	L
**Total**		**62 ± 8.5**	**26 ± 3.7**	**23.5 ± 12.1**	**2.2 ± 1.2**	**29.2 ± 26.5**		

Body mass index (BMI), follow-up (FU), gluteus medius muscle (Gmed), PPI periprosthetic infection (PPI), aseptic loosening (ASL), insufficient muscular refixation (MR), other reasons (O), primary implantation (PI), gluteus muscle transfer (GMT), anterolateral (AL), lateral (L)

### Radiographic results

Overall, the MRI evaluation showed no significant changes in total muscle volume during the follow-up period (*p* = 0.317). Moreover, no significant changes were found for total lean muscle volume (*p* = 0.317) and total fat volume (*p* = 0.317) of the abductor mechanism. Separate measurements were made for the Gmin, Gmed, Gmax and TFL. The lean muscle volume presented a significant growth of muscular tissue for the Gmin (*p* = 0.028) and TFL (*p* = 0.046) compared to the preoperative measurements. The Gmax presented a significant loss of total volume, but no significant results regarding the lean muscle volume. The amount of fat volume decreased for all measured muscles with statistical significance for the Gmin (*p* = 0.028), the Gmed (*p* = 0.046) and the TFL (*p* = 0.028). A comparison of the pre- and postoperative levels of muscle degeneration presented no relevant changes overall according to Goutallier’s classification. Only a mild recovery was seen for the Gmin (G4 to G3) in one case and for the TLF (G3 to G2) in another case. In all other measurements, neither a further degeneration nor a recovery was found. Moreover, the measurement presented a flap survival for all patients, without any signs of flap necrosis (Table 2).

**Table 2:** Patients’ pre- and postoperative radiographic results

	Total (mean ± SD)		
	Preop	Postop	***p*** **-value**
**LeanVol**			
Gmin	22,183 (± 4130)	31,131 (± 5254)	0,028*
Gmed	170,071 (± 65,236)	191,352 (± 69,107)	0,345
Gmax	445,261 (± 180,263)	463,192 (± 275,538)	0,6
TFL	59,993(± 41,276)	70,187 (± 42,527)	0,046*
	697,508 (± 75,971)	755,862 (± 121,152)	0,317
**TotalVol**			
Gmin	42,633 (± 5325)	41,049 (± 6891)	0,249
Gmed	230,259 (± 56,888)	216,009 (± 73,407)	0,463
Gmax	710,379 (± 237,557)	649,282 (± 204,404)	0,046*
TFL	75,173 (± 40,039)	77,548 (± 39,617)	0,6
	1,058,445 (± 103,977)	983,888 (± 86,585)	0,317
**TotalFat**			
Gmin	20,450 (± 5289)	9919 (± 6026)	0,028*
Gmed	60,189 (± 43,022)	24,657 (± 67,345)	0,046*
Gmax	265,118 (± 115,089)	186,089 (± 99,906)	0,345
TFL	15,180 (± 15,781)	7361 (± 13,567)	0,028*
	360,937 (± 49,487)	228,026 (± 44,756)	0,317

*Statistically significant p-value < 0.05. Gluteus minimus muscle (Gmin), gluteus medius muscle (Gmed), gluteus maximus muscle (Gmax), tensor fascia lata (TFL), lean muscle volume, (LeanVol) total muscle volume (TotalVol), fat volume (FatVol)

### Clinical results

All patients presented a positive Trendelenburg sign, a positive internal rotation lag sign, limping and local pain preoperatively. At the time of the latest follow-up, two out of six patients presented a negative Trendelenburg sign and a negative internal rotation lag sign. Greater Trochanteric pain syndrome was recorded in two cases pre- and postoperatively. The average HHS score improved from 38.3 ± 15.8 to 45.3 ± 24.6 postoperatively; however, this was not statistically significant (*p* = 0,345). Postoperatively, the functional status was excellent in two patients, good in one and poor in three patients. Preoperative limping was recorded as severe in five cases and as light in one case. Postoperative limping significantly decreased and was seen as light for three and as moderate for three patients. Four of the six patients reported that they would undergo the procedure again.

The general level of hip pain after the NRS decreased from NRS 7.5 ± 1.5 to 5.1 ± 2.6 postoperatively. The mean abduction power according to Janda’s classification displayed an increase from 1.8 ± 1.0 to 2.5 ± 0.8 postoperatively (Table 3).

**Table 3:** Patients’ preoperative and postoperative functional results (Harris hip score) after gluteus maximus transfer

	Preoperative	Postoperative	*p*-value
Pain	10.0 ± 6.3	14.0 ± 16.5	0,461
Limp	1.3 ± 3.3	6.5 ± 1.6	0,038*
Support	6.0 ± 3.9	3.7 ± 4.1	0,197
Distance	4.5 ± 2.3	5.5 ± 1.2	0,317
Stairs	1.7 ± 0.5	1.8 ± 1.2	0,705
Shoes/socks	2.7 ± 1.0	2.3 ± 0.8	0,317
Sitting	3.5 ± 2.0	2.3 ± 2.0	0,357
Transport	0.3 ± 0.5	0.7 ± 0.5	0,157
Deformity	4.0 ± 0	4.0 ± 0	1
ROM	4.3 ± 0.5	4.5 ± 0.5	0,564
**HSS (total)**	38.3 ± 15.8	45.3 ± 24.6	0,345

*Statistically significant p-value < 0.05. Harris hip score (HHS), range of motion (ROM)

### Complications

No infection or dislocation was detected. No further revision was required during the follow-up period.

## Discussion

The gluteus maximus flap transfer is an exceptional surgical procedure for patients with chronic abductor mechanism deficiency of the hip joint. Patients present different medical histories including several prior hip surgeries. The present case series indicates that the Gmax flap transfer offers promising results regarding pain, limping, muscle volume, radiographic flap survivor rate and patient satisfaction for affected patients. Although the good to excellent clinical results of the published data could not be confirmed in this present study [14, 18]. Whiteside and Roy suggested that gluteus maximus flap transfer might improve patient outcomes after total hip arthroplasty with acetabular bone loss [14]. Another study by Maldonado et al. reported of significant improvements in patient-reported outcomes after gluteus maximus flap transfer and transfer of the tensor fasciae latae due to irreparable full thickness ruptures of the gluteus medius [26]. These findings are in line with good subjective results of the present study.

To the best of our knowledge, this study presents the first cohort of patients treated with a Gmax transfer after THA focusing on pre- and postoperative radiographic MRI evaluation and flap survival.

Several advantages of the Gmax transfer are reported compared to other surgical techniques [18, 20]. It is a feasible option even in the case of bone loss, as the flap can support a deficiency of the posterior capsule and the origin as well as the insertion of the advanced muscle is not affected [18, 21]. Moreover, it can be effective in the case of implant instability and is recommended for complete Gmed tears with a gap extension up to 10 centimeters [18, 21]. The disadvantages are reported as follows: An experienced surgeon is mandatory, the restoration of active abduction is poor, the force vector for abduction is abnormal after the treatment and the results are related to the interposition effect [18, 21].

### Clinical findings

The present study presents fair functional results after Gmax transfer. Overall, no significant clinical postoperative improvements regarding the HHS were found. Moreover, two-thirds of the patients presented unmodified positive Trendelenburg and internal rotation lag signs at the last follow-up. Postoperative improvements were found regarding limping. These findings partly contrast those of the published literature. The excellent results published by Whiteside and Roy were not reproducible in the present study [14]. This study reviewed the medical records of 18 patients who underwent this procedure and found significant improvements in pain and function scores postoperatively. Excellent postoperative results with the absence of limping and pain as well as a negative Trendelenburg sign for a majority of the included patients. These different results might be caused by the inhomogeneous and small patient cohorts in both studies. In addition, it cannot be ruled out that the gluteus maximus transfers was performed more accurate in the study of Whiteside due to their greater experience with this specific surgical procedure. Moreover, another systematic review by Odak and Ivory indicated that functional improvements after surgical treatment strongly depend on the initial amount of muscular damage [5]. A study by Ricciardi et al. treated an inhomogeneous cohort of seven patients with a history of previous hip surgeries with a Gmax flap transfer due to soft tissue deficiency [21]. Their functional results regarding postoperative pain, Trendelenburg sign and limping are less enthusiastic and similar to the findings of the present study [21]. A recently published study with a cohort of 15 patients included even reported of worse functional results after gluteus maximus flap transfer [27]. No improvements in the hip abductor moment was measured. These results underline the difficulty of the treatment of chronic hip abductor deficiency [27]. Reflecting on all these studies, a relevant gap between satisfying subjective and sobering objective results can be seen. Patients might not want to admit their persistent functional limitation.

Besides fair functional results, the soft tissue coverage was pointed out as particularly useful protection against pain but also to prevent local seroma or hematoma cavities [21]. However, problems regarding soft tissue coverage were not the focus of the present study and did not play a considerable role pre- and postoperatively.

### Radiographic findings

**Fig. 3:**
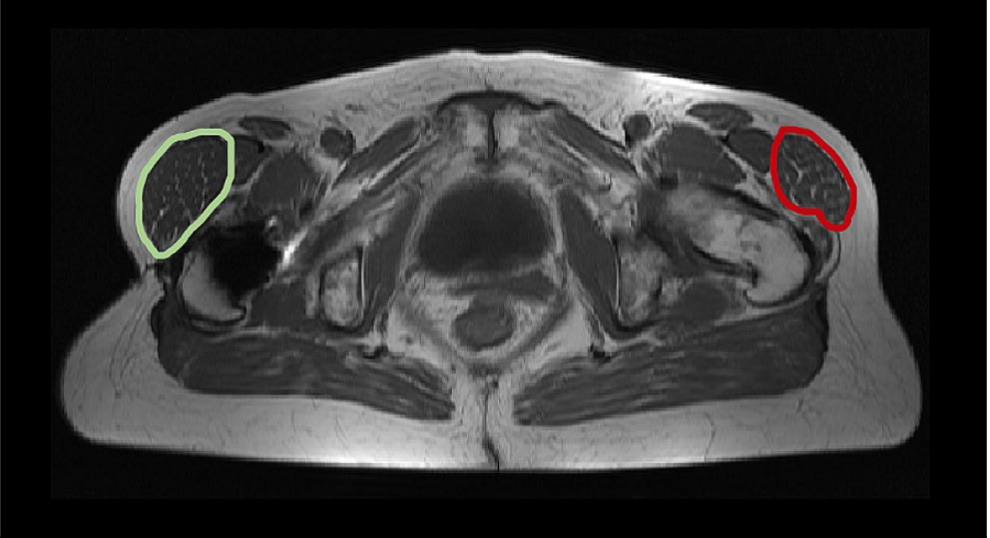
Example of postoperative measurements compared to the unaffected side. Postoperative MRI of the pelvis (T1, transversal plane) of a 56-year-old patient with a chronic gluteal deficiency of the right abductor mechanism after total hip arthroplasty treated with a gluteus maximus transfer. The MRI images 11 months after surgery presenting a compensatory growth of the right musculus tensor facia lata (green) compared to the opposite and unaffected left side (red)

The present study shows satisfying results regarding the postoperative muscle volume. Despite the invasiveness of the procedure, overall no significant further loss of total muscle volume was seen. Due to the progressive nature of muscular degeneration, these findings can be interpreted as treatment success. The detailed results present some remarkable changes in muscle tissue that have to be discussed. A trend toward a compensatory growth of the TFL volume can be seen postoperatively. The Gmed as the strongest abductor with the highest level of degeneration preoperatively demonstrated no significant further loss of total volume during the follow-up period. The moderate total volume loss of Gmax is explainable due to the invasive operative procedure. The measurements of the present study indicate that no further loss of the Gmax muscle volume can be verified, despite the part used and elevated as a flap. The lean muscle volume exhibited better postoperative results for all measured muscles, indicating a postoperative growth of functional muscle tissue of the abductor mechanism. In particular, the significant growth of the Gmin and the TFL has to be pointed out. It can be assumed that abduction-supportive muscles such as the TFL grow as a compensation (Fig. 3). A study of Damm et al. with a short-term follow-up of three months after THA also reported a significant increase in TFL lean volume and a decreased Gmin volume postoperatively [28]. A decreased Gmin volume contrasts the findings of the present study and can be explained by the largely different follow-up period. However, the TFL seems to play an important role to compensate for the loss of the gluteal muscles during THA.

The general growth of functional muscle tissue (lean volume) may be a result of the reduced reliving posture of the affected hip and result clinically as an increased level of limping.

Furthermore, the muscles of the abductor mechanism exhibited no significant differences in degeneration according to the Goutallier classification. The level of fatty degeneration of the Gmed muscle seemed to be reduced, although the reasons for that are unknown. A lack of further degeneration of the Gmed muscle after the procedure can be viewed as a success. The effect on the Gmax muscle throughout the preparation and elevation of the flaps has not led to a measurable further degeneration or a significant loss of its size.

### Limitations

The present study has several limitations. First, this study comprises a small number of patients. Second, this study is limited due to the inhomogeneous study population with various different numbers and reasons of prior hip surgeries and a wide range in terms of patients’ age. This results in a reduced comparability of the patients. Third, different follow-up periods are limiting this study and fourth, the measurement accuracy is marginal and limited in some image slices due to the changed postoperative configuration of muscular tissue. Fourth, radiographic measurements only considered overall muscle volumes. To draw conclusion based on these measurements regarding specific muscle function might be inexact.

## Conclusions

Reflecting on the functional, subjective and radiographic results of this present study, the Gmax flap transfer presents optimistic results for patients with a history of frustrating conservative treatment and advanced degeneration of the abductor mechanism. Besides fair functional results, patients reached satisfying postoperative radiographic results. No further degeneration, loss in muscle volume or flap necrosis were measurable. The results of the current study indicate that the surgical procedure may result in an increase in limping and a subsequent growth of functional muscular tissue following the operation. However, it is important to note that the surgical treatment of abductor mechanism deficiency following total hip arthroplasty continues to pose challenges in terms of treatment options for both patients and surgeons. Further prospective studies with larger and more homogeneous patient cohorts are needed to confirm these findings.

## Data Availability

The data presented in this study are available on request from the corresponding author. The data are not publicly available due to patients’ data protection.
